# Prescribing characteristics and guideline concordance of antihypertensive western and Chinese patent medicine in Internet hospitals in China: a cross-sectional study

**DOI:** 10.3389/fphar.2025.1580787

**Published:** 2025-04-28

**Authors:** Tiantian Zhou, Xing Liao, Jiaxin Zuo, Fang Han, Ruogu Meng, Lin Zhuo, Guozhen Liu, Jing Hu

**Affiliations:** ^1^ Beijing Evidence-based Chinese Medicine Center , Beijing Hospital of Traditional Chinese Medicine, Capital Medical University, Beijing Institute of Chinese Medicine, Beijing, China; ^2^ Center for Evidence-based Medicine, Institute of Basic Research in Clinical Medicine, China Academy of Chinese Medical Sciences, Beijing, China; ^3^ National Institute of Health Data Science, Peking University, Beijing, China; ^4^ Research Center of Clinical Epidemiology, Peking University Third Hospital, Beijing, China; ^5^ Beijing PD Cloud medical Technology Co., Ltd., Beijing, China

**Keywords:** hypertension, Internet hospitals, Chinese patent medicine, Western medicine, prescribing characteristics, guidelines concordance

## Abstract

**Introduction:**

Internet hospitals have emerged as a vital approach for patients seeking treatment for hypertension, with a significant increase in antihypertensive medication prescriptions through these innovative models. However, prescribing characteristics and guideline concordance of these prescriptions remain unclear. This study aim to analyze the prescribing characteristics of Western medicine (WM) and Chinese patent medicine (CPM) for hypertension and assess their concordance with hypertension guidelines in Internet hospitals in China, providing insights for optimizing antihypertensive CPM management.

**Methods:**

A cross-sectional analysis was conducted using data from the Yinchuan Internet Medical Regulatory Platform (YIMRP) covering 87 enterprise-led Internet hospitals in China from 1 January 2018, to 31 March 2021. Visits diagnosed with hypertension and prescribed at least one oral antihypertensive medication (either WM or CPM) were included. Guideline concordance was evaluated by comparing prescribed individual antihypertensive WM and CPM in Internet hospitals with international and Chinese hypertension guidelines recommendations. Statistical analyses included descriptive statistics, association rule analysis, and guideline concordance assessment.

**Results:**

Among the 787,209 visits, 93.75% were prescribed WM alone, 4.72% CPM alone, and 1.52% a combination of CPM and WM. Calcium channel blockers (CCBs) (38.50%) was the most prescribed WM class, with nifedipine (19.67%) being the most common individual medication. Most prescriptions of antihypertensive WM were guideline-concordant. Among CPM prescriptions, only 181 (0.37%) included traditional Chinese medicine (TCM) syndrome diagnoses. Of the 38 prescribed antihypertensive CPM, only 7 were guideline-recommended. The most frequently prescribed CPM were Jiuqiang Naoliqing (17.67%), and Zhenju Jiangya tablet (14.74%), neither of which was recommended by the guidelines. The combinations of two CPM were frequently prescribed, but none of these combinations were recommended by guidelines. The most common dual CPM combination was Jiuqiang Naoliqing + Qiangli Dingxuan tablet/capsule (*support* 8.65%, *confidence* 0.44%).

**Conclusion:**

The prescribing characteristics of antihypertensive WM in Internet hospitals closely align with those in offline hospitals with relatively satisfactory guideline concordance. However, some issues persist in antihypertensive CPM prescriptions, including the lack of TCM syndrome differentiation, frequent prescription of non-recommended CPM, and duplicate therapies. Strengthening CPM management in Internet hospitals is essential for optimizing hypertension care.

## 1 Introduction

Hypertension, is a leading cause of cardiovascular disease (CVD) and premature death, contributing to 226 million disability-adjusted life years (DALYs) and 10.9 million deaths worldwide in 2021 (Zhang H. et al., 2024a). In China, the prevalence of hypertension has increased from 27.5% in 2018 to 31.6% in 2022 (National Center for Cardiovascular Diseases, 2024). To alleviate the burden of hypertension, the Chinese government has implemented comprehensive intervention strategies, including the *Healthy China Action Plan* (2019–2030), with the aim of ensuring that at least 70% of hypertensive patients receive standardized management by 2025 (General Office of the State Council of China, 2017; National Health Commission, 2019).

Numerous guidelines for the management of hypertension have been developed globally and are widely adopted by clinicians as essential reference standards to optimize patient care (Whelton et al., 2018; McEvoy et al., 2024; Zhu et al., 2024). In China, the government has issued a series of policy documents and guidelines on hypertension management such as *Chinese Guideline for the Prevention and Treatment of Hypertension*, *Guideline on the Clinical Application of Chinese Patent Medicine in the Treatment of Hypertension*, and *Expert Consensus on Chinese Medicine Diagnosis and Treatment of Hypertension*, which recommended both Western medicine (WM) and Chinese patent medicine (CPM) for hypertension treatment (Society of Cardiovascular Diseases, 2019; Xu, 2022; Wang, 2024). For antihypertensive WM, angiotensin-converting enzyme inhibitors (ACEIs), angiotensin receptor blockers (ARBs), calcium channel blockers (CCBs), and diuretics are recommended as the initial treatment options for hypertension in international and Chinese guidelines (Whelton et al., 2018; Writing Group of Chinese Guidelines for the Management of Hypertension et al., 2019; McEvoy et al., 2024). Additionally, Chinese guidelines emphasize the use of syndrome-specific CPM, such as Songling Xuemaikang capsule, Tianma Gouteng granule, and Qinggan Jiangya capsule, for tailored hypertension management (Xu, 2022; Wang, 2024).

Adherence to guideline recommendations has been demonstrated to enhance blood pressure control and reduce cardiovascular risks (Paturle et al., 2023; Hias et al., 2024). However, international guideline concordance remains suboptimal in clinical practice, preventing many patients from obtaining the full therapeutic benefit of evidence-based interventions (Vatani Nezafat et al., 2020; Harrison et al., 2023). Patients with poor blood pressure control due to guideline non-adherence have significantly increased risks of stroke and myocardial infarction (Colantonio et al., 2018). Furthermore, persistent blood pressure variability accelerates the progression of atherosclerosis and increases susceptibility to target organ damage, particularly renal failure and retinopathy (Saputra et al., 2024; Buffolo et al., 2021). These clinical consequences of guideline discordance ultimately lead to suboptimal hypertension management outcomes and impose increased economic burdens on healthcare systems (Alyabsi et al., 2020). This highlights the importance of adhering to guidelines to assist physicians in delivering evidence-based and appropriate treatments for hypertensive patients (Weber et al., 2014; Langford et al., 2020).

Internet hospitals, an emerging healthcare delivery model, provide a comprehensive range of online medical services, including appointment scheduling, online consultations, electronic prescribing, medication delivery, health education, and follow-up care for chronic disease (Xu et al., 2021b; Chen et al., 2023). The first Internet hospital base in China was established on 19 March 2017, in Yinchuan city (Xie et al., 2017). Since then, Internet hospitals have rapidly expanded nationwide, significantly improving healthcare access for both patients and providers (Han et al., 2020). One of the most notable advantages of this model is its ability to reduce time and cost for patients, making it particularly beneficial for individuals with hypertension who require long-term medication management. Internet hospitals facilitate prescription renewals and ensure timely access to medications, addressing critical needs for these patients (Zhang et al., 2020; Zhang, 2022). With the continued development of Internet hospitals, an increasing number of patients are seeking hypertension treatment online, resulting in a growing trend of antihypertensive medication prescriptions through these platforms (Gao et al., 2024). In contrast to traditional offline hospitals, Internet hospitals are primarily reliant on patient-reported data (e.g., self-measured blood pressure), subjective symptom descriptions, and retrospective medical records, with additional constraints including the unavailability of physical examinations and limited capacity for Traditional Chinese Medicine (TCM) syndrome differentiation. These challenges are further exacerbated by heterogeneous clinical training and institutional backgrounds among physicians on enterprise-led platforms, ultimately resulting in disparities in prescribing behaviors and guideline adherence between Internet-based and traditional offline healthcare systems (Tang et al., 2024).

Previous studies have extensively investigated the prescription patterns of antihypertensive medications and their adherence to clinical guidelines in offline hospitals and primary healthcare settings in China (Su et al., 2017; Yang et al., 2023; Lu et al., 2024). However, data on these practices in Internet hospitals remain scarce. Therefore, this study aims to analyze the prescribing characteristics of antihypertensive medications in Internet hospitals, encompassing both WM and CPM, and evaluate their concordance with hypertension guidelines. The findings will provide valuable insights to optimize antihypertensive medication management in this emerging healthcare model in China.

## 2 Materials and methods

### 2.1 Data source and study subjects

Data were extracted from the Yinchuan Internet Medical Regulatory Platform (YIMRP), established in 2017 by the Yinchuan Municipal Government. This platform integrates all enterprise-led Internet hospitals in Yinchuan and covers approximately 80% of such hospitals nationwide (Wang, 2022). Patients utilizing these Internet hospitals are distributed across China, representing a broad and diverse population base (People’s Government of Yinchuan City, 2018). The Internet hospital code, patient’s ID, patient age and gender, visiting departments, disease diagnosis, prescription date, name and cost of prescription medication, and TCM syndrome are stored in this platform. Data extraction and analysis in this platform are conducted routinely every 3–4 years.

**FIGURE 1:**
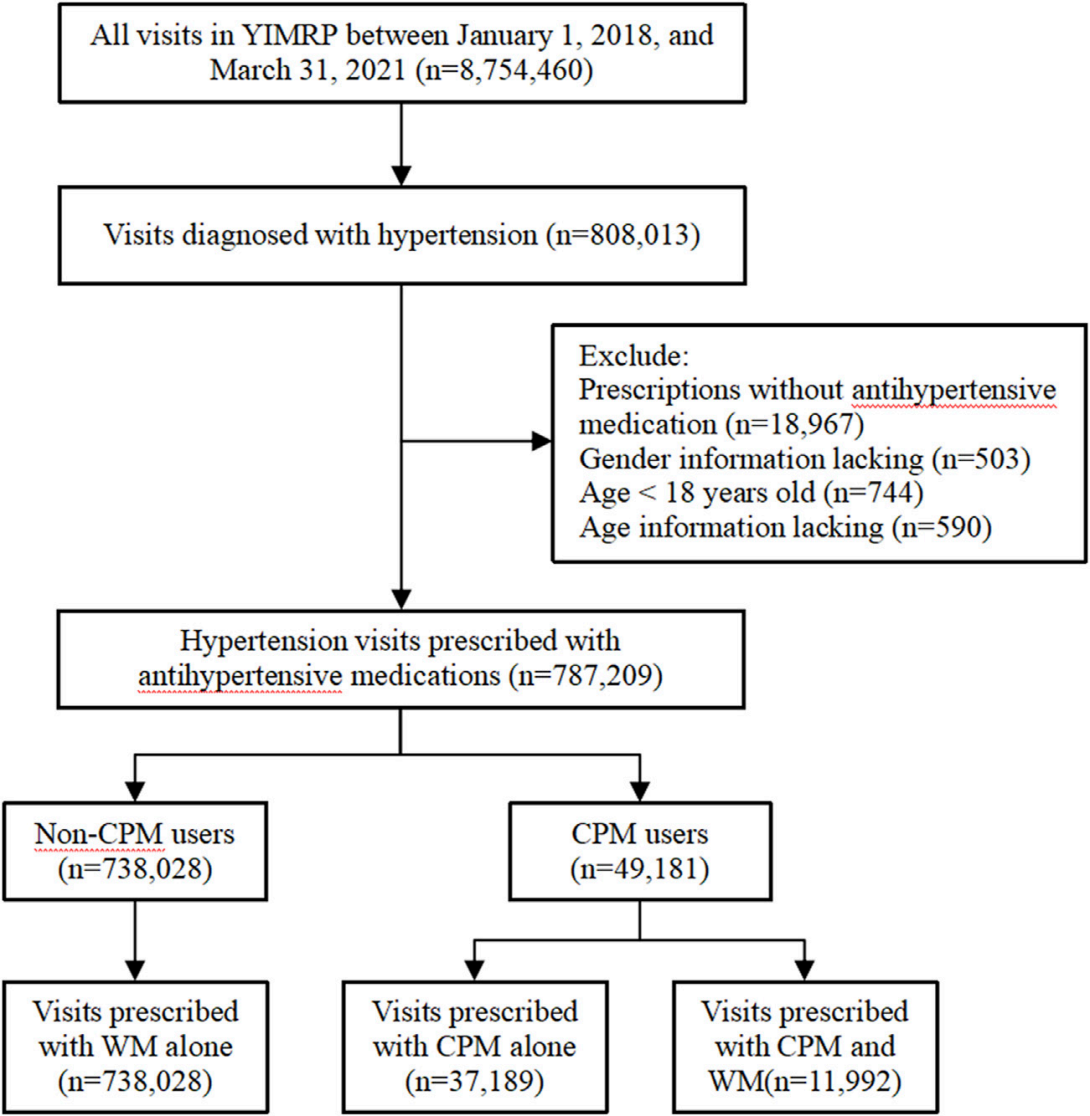
Flow chart of screening eligible visits.

This study conducted a cross-sectional analysis using data from YIMRP, which recorded a total of 8,754,460 visits across 87 Internet hospitals between 1 January 2018 and 31 March 2021. The study included all patients diagnosed with hypertension during this period. Exclusion criteria included: 1) patients who were not prescribed any oral antihypertensive medication (including neither WM nor CPM). 2) patients aged under 18 years, and 3) patients with missing age or gender data. After applying these exclusion criteria, the final analytical sample consisted of 787,209 visits. A flowchart detailing the screening process for eligible visits is presented in Figure 1. This study followed the Strengthening the Reporting of Observational Studies in Epidemiology (STROBE) reporting guideline (von Elm et al., 2008).

### 2.2 Data management

Disease diagnoses were coded according to the International Classification of Diseases, version 10 (ICD-10). Comorbidities among hypertensive patients were identified based on diagnostic information associated with their prescriptions. WM recorded by brand names in the database were initially converted to their generic names. Subsequently, each antihypertensive WM was classified as ACEIs, ARBs, CCBs, diuretics, and other classes based on the Anatomical Therapeutic Chemical (ATC) fifth classification system (World Health Organization, 2022). CPM were standardized according to the *Chinese pharmacopoeia (2020 edition)* (Chinese Pharmacopoeia Commission, 2020) and unified under their official names. Since CPM were not included in ATC classification system, formulations with identical active ingredients but different dosage forms were consolidated into single categories. These categories were further grouped by their TCM efficacy according to *China’s National Reimbursement Drug List* (National Health Commission, 2023). Each category was mutually exclusive, ensuring that no CPM could be classified into more than one group.

### 2.3 Guideline concordance

Given that the study data spanned from 2018 to 2021, we reviewed contemporaneous international and Chinese hypertension guidelines. The influential *2017 American College of Cardiology and the American Heart Association (ACC/AHA) hypertension guideline* (Whelton et al., 2018) and the *2018 European Society of Cardiology and European Society of hypertension (ESC/ESH) Guideline* (Williams et al., 2018) served as primary reference standards for WM evaluation (Kow et al., 2021). As our data came from Chinese Internet hospitals, we also referenced the *2018 Chinese Guideline for the Prevention and Treatment of Hypertension* (Writing Group of Chinese Guidelines for the Management of Hypertension et al., 2019), which incorporated evidence from Chinese population studies.

Since these guidelines lacked CPM recommendations, we further referenced two authoritative Chinese hypertension guidelines to evaluate the guideline concordance of CPM: *2021 Guideline on the Clinical Application of Chinese Patent Medicine in the Treatment of Hypertension* (Xu, 2022), and the *2019 Expert Consensus on Chinese Medicine Diagnosis and Treatment of Hypertension* (Society of Cardiovascular Diseases, 2019).

Medications prescribed in Internet hospitals demonstrate guideline concordance if they are recommended by any of the aforementioned guidelines. Conversely, they are considered guideline-discordant if they are either explicitly not recommended or not mentioned in these guidelines. The guideline-recommended medications are detailed in Supplementary Tables S1, S2.

### 2.4 Statistical analysis

Continuous variables, such as age, were expressed as mean and standard deviation (SD), while categorical variables, including gender, age group, and comorbidities, were presented as frequencies and percentages. All visits were categorized into three groups: the CPM group, the WM group, and the CPM combined with WM group. Differences in continuous variables across the three groups were analyzed using Analysis of Variance (ANOVA), while differences in categorical variables were assessed using Pearson’s chi-square tests. Age was stratified into four categories: 18–44 years, 45–59 years, 60–74 years, and ≥75 years. All statistical tests were two-sided, with P-value <0.05 considered statistically significant. Data analyses were performed using SAS for Windows (version 9.4; Order Number: 9C1XJD). To evaluate medication combinations for hypertension, association rule analysis and network visualization were conducted using IBM SPSS Modeler software (version 18.0). The minimum support and confidence thresholds were set to 1, with weak links defined as ≤15 and strong links as ≥35 in the association rules diagram. Sunburst diagrams were performed using WPS Office 2024.

## 3 Results

### 3.1 Characteristics of eligible visits

Among the 787,209 hypertension visits, 738,028 (93.75%) were prescribed WM alone, 37,189 (4.72%) prescribed CPM alone and 11,992 (1.52%) prescribed CPM combined with WM. Female patients were more likely to be prescribed antihypertensive CPM alone. Clinicians tended to prescribe CPM alone for patients aged <45 years, WM alone for those aged 45–59 years, and a combination of CPM and WM for patients aged ≥60 years. Regarding comorbidities, patients with diabetes or angina were more likely to be prescribed WM alone, whereas those with upper respiratory tract infection, migraine, gastrointestinal ulcer, or urinary tract infection were more likely to be prescribed CPM alone. Notably, patients with cardiovascular comorbidities, including coronary heart disease, hyperlipidemia, cerebral infarctions sequelae, and arrhythmia, were more frequently prescribed combinations of CPM and WM. The demographic characteristics of the eligible visits are presented in Table 1.

**TABLE 1:** Demographic characteristics of eligible visits.

Characteristics	WM alone n = 738,028 (%)	CPM alone n = 37,189 (%)	CPM combined with WM n = 11,992 (%)	*P* Value
Gender				<0.001
Male	456,448 (61.85)	22,199 (59.69)	7,598 (63.36)	
Female	281,580 (38.15)	14,990 (40.31)	4,394 (36.64)	
Age (y), Mean ± SD	54.07 ± 12.62	53.96 ± 12.59	55.84 ± 12.45	<0.001
Age(y)				<0.001
18–44	169,852 (23.01)	8,832 (23.75)	2,227 (18.57)	
45–59	324,823 (44.01)	16,121 (43.35)	5,210 (43.45)	
60–74	198,071 (26.84)	9,947 (26.75)	3,620 (30.19)	
≥75	45,282 (6.14)	2,289 (6.15)	935 (7.80)	
Comorbidities				
Coronary heart disease	114,916 (15.57)	5,279 (14.20)	2,998 (25.00)	<0.001
Upper respiratory tract infection	45,445 (6.16)	2,875 (7.73)	551 (4.59)	<0.001
Hyperlipidemia	37,652 (5.10)	1,239 (3.33)	622 (5.19)	<0.001
Diabetes	33,789 (4.58)	1,210 (3.25)	519 (4.33)	<0.001
Angina	26,001 (3.52)	313 (0.84)	413 (3.44)	<0.001
Migraine	13,808 (1.87)	1,707 (4.59)	451 (3.76)	<0.001
Gastrointestinal ulcer	14,296 (1.94)	789 (2.12)	168 (1.40)	<0.001
Cerebral infarctions sequelae	11,579 (1.57)	1,150 (3.09)	617 (5.15)	<0.001
Arrhythmia	11,427 (1.55)	281 (0.76)	233 (1.94)	<0.001
Urinary tract infection	7,491 (1.02)	466 (1.25)	97 (0.81)	<0.001

### 3.2 Prescribing characteristics and guideline concordance of antihypertensive WM

**FIGURE 2:**
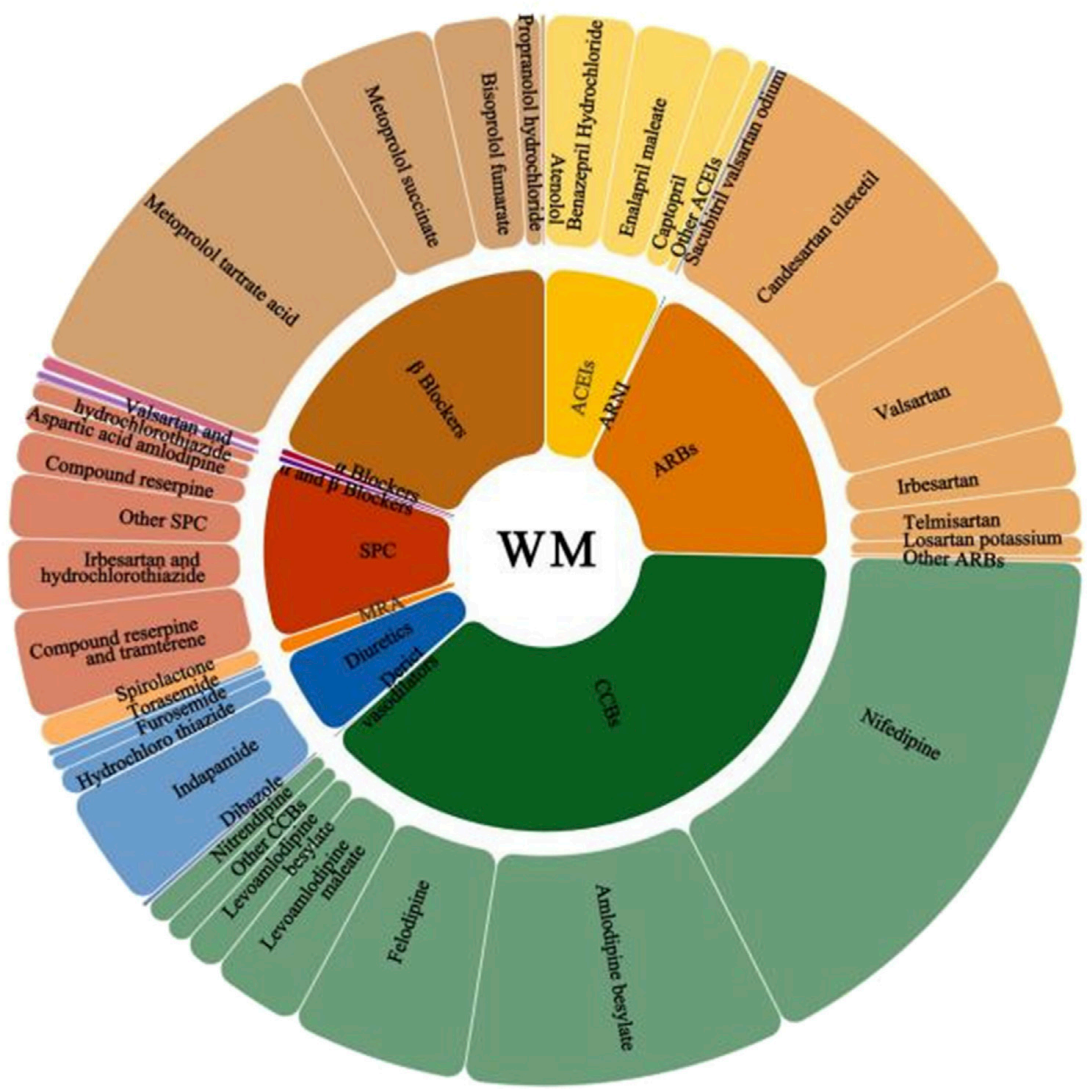
Individual and classes of all antihypertensive WM. Note: ACEIs, angiotensin-converting enzyme inhibitors; ARNI, angiotensin receptor-neprilysin inhibitor; ARBs, angiotensin receptor blockers; CCBs, calcium channel blockers; SPCs, single-pill combinations; WM with a frequency less than 0.5% in a category are grouped as “other”.

A total of 11 classes of antihypertensive WM were prescribed, encompassing 72 individual medications (Figure 2). The most frequently prescribed classes were CCBs (n = 345,733, 38.50%), beta-blockers (n = 171,174, 19.06%), ARBs (n = 164,460, 18.31%) and single-pill combinations (SPCs) (n = 89,282, 9.94%). The most commonly prescribed individual medications were nifedipine (n = 154,809, 19.67%), metoprolol tartrate (n = 101,689, 12.92%), and amlodipine besylate (n = 89,064, 11.31%). A total of 94.58% (n = 744,542) of WM prescriptions were concordant with guidelines. Table 2 summarizes the first-line classes of antihypertensive WM (ACEIs, ARBs, CCBs, and diuretics) and individual medications prescribed in Internet hospitals and their concordance with guidelines, all of these medications were guideline-recommended therapies for hypertension management. The guideline concordance of the remaining seven classes of antihypertensive WM is detailed in Supplementary Table S3. Among these, five classes were widely prescribed and recommended in the Chinese guideline, including SPCs, mineralocorticoid receptor antagonists (MRAs), alpha and beta-blockers, alpha-blockers, and beta-blockers.

**TABLE 2:** Individual and classes of first-line antihypertensive WM in Internet hospitals and guideline concordance.

Internet hospitals			Guidelines		
Class	Individual WM	Prescription frequency (n, %)	2017 ACC/AHA guideline	2018 ESC/ESH guideline	2018 Chinese guideline
ACEIs	Benazepril hydrochloride	24,890 (3.16)	✓	✓	✓
	Enalapril maleate	21,609 (2.75)	✓	✓	✓
	Captopril	11,329 (1.44)	✓	✓	✓
	Fosinopril sodium	2,644 (0.34)	✓	✓	✓
	Perindopril	1,046 (0.13)	✓	✓	✓
	Lisinopril	485 (0.06)	✓	✓	✓
	Imidapril hydrochloride	170 (0.02)	✕	✓	✓
	Ramipril	116 (0.01)	✓	✓	✓
	Benazepril	12 (0.00)	✓	✓	✓
ARBs	Candesartan cilexetil	83,732 (10.64)	✓	✓	✓
	Valsartan	42,685 (5.42)	✓	✓	✓
	Irbesartan	17,350 (2.20)	✓	✓	✓
	Telmisartan	13,465 (1.71)	✓	✓	✓
	Losartan potassium	5,693 (0.72)	✓	✓	✓
	Olmesartan medoxomil	1,142 (0.15)	✓	✓	✓
	Allisartan lsoproxil	393 (0.05)	✕	✓	✓
CCBs	Nifedipine	154,809 (19.67)	✓	✓	✓
	Amlodipine besylate	89,064 (11.31)	✓	✓	✓
	Felodipine	48,260 (6.13)	✓	✓	✓
	Levoamlodipine maleate	25,037 (3.18)	✕	✓	✓
	Levoamlodipine Besylate	10,913 (1.39)	✕	✓	✓
	Nitrendipine	7,154 (0.91)	✕	✓	✓
	Amlodipine maleate	2743 (0.35)	✓	✓	✓
	Lacidipine	2,248 (0.29)	✕	✓	✓
	Benidipine	2,010 (0.26)	✕	✓	✓
	Lercanidipine	1,324 (0.17)	✕	✓	✓
	Diltiazem	1,165 (0.15)	✓	✓	✓
	Amlodipine mesylate	573 (0.07)	✓	✓	✓
	Cilnidipine	204 (0.03)	✕	✓	✓
	Manidipine	188 (0.02)	✕	✓	✓
	Amlodipine	40 (0.01)	✓	✓	✓
	Nicadipine	1 (0.00)	✕	✓	✓
Diuretics	Indapamide	33,903 (4.31)	✓	✓	✓
	Hydrochlorothiazide	7,962 (1.01)	✓	✓	✓
	Furosemide	4,514 (0.57)	✕	✓	✓
	Torasemide	1,953 (0.25)	✕	✓	✓

Note: ✓: Concordance with guideline recommendations; ✕: Discordant with guideline recommendations; ACEIs, angiotensin-converting enzyme inhibitors; ARBs, angiotensin receptor blockers; CCBs, calcium channel blockers.

**FIGURE 3:**
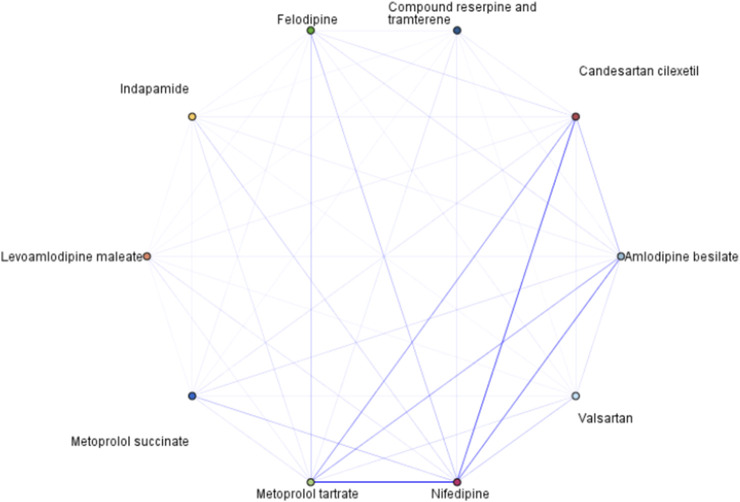
Association rule diagram of antihypertensive WM combinations. Note: Weak links ≤15 and strong links ≥35. The stronger line showed more frequent co-prescribed between medicine.

Among the antihypertensive WM combinations, dual therapy was the most common (Figure 3; Table 3). The most common combination was nifedipine + metoprolol tartrate (*support* 18.65%, *confidence* 8.43%), followed by nifedipine + candesartan cilexetil (*support* 18.65%, *confidence* 6.74%). Both combinations align with guideline recommendations, which endorse the use of a CCB in combination with either a beta-blocker or an ARB. In contrast, combinations involving two CCBs, such as nifedipine + amlodipine besylate (*support* 18.65%, *confidence* 5.87%), felodipine + nifedipine (*support* 6.02%, *confidence* 10.18%), and felodipine + amlodipine besylate (*support* 6.02%, *confidence* 5.92%), were not recommended in any of the guidelines.

**TABLE 3:** Commonly prescribed antihypertensive WM combinations and guideline concordance.

Internet hospitals					Guidelines		
Prescribed WM combination	Classes	Support (%)	Confidence (%)	Lift	2017 ACC/AHA guideline	2018 ESC/ESH guideline	2018 Chinese guideline
Dual therapy							
Nifedipine + Metoprolol tartrate	A + B	18.65	8.43	0.68	✓	✓	✓
Nifedipine + Candesartan cilexetil	A + C	18.65	6.74	0.65	✓	✓	✓
Nifedipine + Amlodipine Besylate	A + A	18.65	5.87	0.53	✕	✕	✕
Metoprolol tartrate + Candesartan cilexetil	B + C	12.46	7.25	0.7	✕	✓	✓
Metoprolol tartrate + Amlodipine Besylate	B + A	12.46	6.59	0.6	✓	✓	✓
Candesartan cilexetil + Amlodipine Besylate	C + A	10.99	6.07	0.59	✓	✓	✓
Felodipine + nifedipine	A + A	6.02	10.18	0.55	✕	✕	✕
Felodipine + Metoprolol tartrate	A + B	6.02	8.39	0.67	✓	✓	✓
Felodipine + Candesartan cilexetil	A + C	6.02	6.81	0.66	✓	✓	✓
Felodipine + Amlodipine Besylate	A+ A	6.02	5.92	0.54	✕	✕	✕
Triple therapy							
Nifedipine + Metoprolol tartrate + Candesartan cilexetil	A + B + C	1.57	5.13	0.5	✕	✓	✓
Nifedipine + Metoprolol tartrate + Amlodipine Besylate	A + B+ A	0.82	6.18	0.33	✕	✕	✕
Candesartan cilexetil + Amlodipine Besylate + Metoprolol tartrate	C+ A + B	0.67	5.65	0.45	✕	✓	✓
Candesartan cilexetil + Amlodipine Besylate + nifedipine	C + A + A	0.67	5.39	0.29	✕	✕	✕
Felodipine + nifedipine + Metoprolol tartrate	A + A + B	0.61	5.08	0.41	✕	✕	✕
Felodipine + nifedipine + Candesartan cilexetil	A + A + C	0.61	3.75	0.36	✕	✕	✕
Felodipine + nifedipine + Metoprolol tartrate	C + A + B	0.60	5.83	0.47	✕	✓	✓
Felodipine + nifedipine + Amlodipine Besylate	C + A + A	0.60	3.97	0.36	✕	✕	✕
Candesartan cilexetil + Metoprolol tartrate + nifedipine	C+ B + A	0.59	4.29	0.42	✕	✓	✓
Candesartan cilexetil + Metoprolol tartrate + Felodipine	C + B + A	0.51	6.28	0.61	✕	✓	✓

Note: A, CCBs; B, Beta-blockers; C, ARBs; ✓: Concordance with guideline recommendations; ✕: Discordant with guideline recommendations.

In the analysis of triple therapy with WM, the combination of a CCB, beta-blocker, and ARB was the most frequently prescribed and was guideline-recommended. Among these combinations, the nifedipine + metoprolol tartrate + candesartan cilexetil (*support* 1.57%, *confidence* 5.13%) was the most commonly prescribed, candesartan cilexetil + amlodipine besylate + metoprolol tartrate (*support* 0.67%, *confidence* 5.65%) was the third most frequent combination. Notably, the guidelines did not recommend combinations involving dual CCBs with either a beta-blocker or an ARB, such as nifedipine + metoprolol tartrate + amlodipine besylate (*support* 0.82%, *confidence* 6.18%), and candesartan cilexetil + amlodipine besylate + nifedipine (*support* 0.67%, *confidence* 5.39%) (Table 3).

### 3.3 Prescribing characteristics and guideline concordance of antihypertensive CPM

**FIGURE 4:**
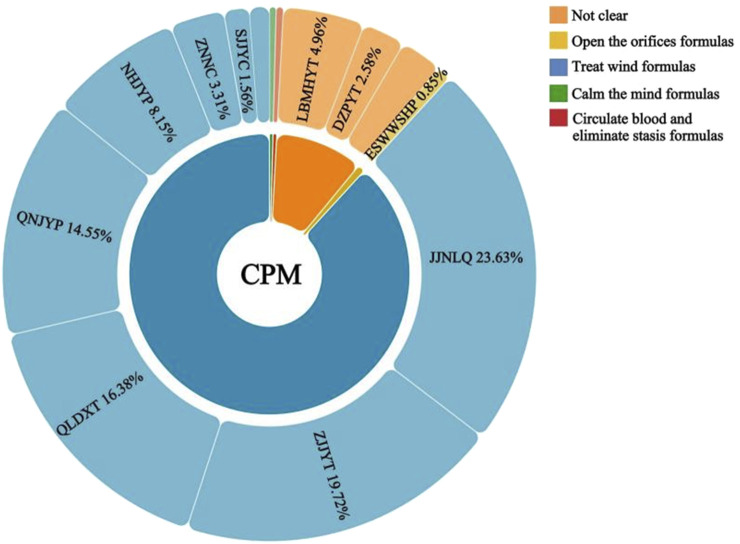
Categories of antihypertensive CPM and the top 10 most common CPM. Note: JQNLQ, Jiuqiang Naoliqing; ZJJYT, Zhenju Jiangya tablet; QLDXT, Qiangli Dingxuan tablet/capsule; QNJYT, Qingnao Jiangya tablet; NHJYP, Niuhuang Jiangya tablet/pill; LBMJYT, Luobuma Jiangya tablet; ZNNC, Zhennaoning capsule/granule; DZPYT, Duzhong Pinya tablet; SJJYC, ShanJu Jiangya capsule; ESWWSHP, Ershiwuwei Shanhu pill.

A total of 38 antihypertensive CPM were prescribed (Supplementary Table S4), they were classified into 4 TCM categories based on their TCM efficacy (Figure 4; Table 4). Descriptions of the TCM categories are detailed in Supplementary Table S5. The most frequently category was treat wind formulas (n = 32,544, 88.51%). The components, TCM syndromes and efficacy of the top 10 prescribed antihypertensive CPM are detailed in Supplementary Table S6. It is worth noting that only 181 prescriptions (0.37%) included TCM syndrome diagnoses for CPM users, due to the limited availability of TCM syndrome data, we did not perform an analysis of TCM syndromes in this study.

**TABLE 4:** Individual and categories of antihypertensive CPM in Internet hospitals and guideline concordance.

Internet hospitals				Guidelines	
TCM category (n, %)	TCM efficacy	Individual CPM (Pin-yin name)	Prescription frequency (n, %)	2021 CPM guideline	2019 hypertension expert consensus
Treat wind formulas (32,544, 88.51)	Soothe the liver and submerge yang	Zhenju Jiangya tablet	7,251 (14.74)	✕	✕
		Qiangli Dingxuan tablet/capsule	6,024 (12.25)	✓	✕
		Qingnao Jiangya tablet	5,348 (10.87)	✓	✕
		Niuhuang Jiangya tablet/pill	2,996 (6.09)	✓	✕
		Zhennaoning capsule/granule	1,218 (2.48)	✕	✕
		Songling Xuemaikang capsule	309 (0.63)	✓	✓
		Tianma Gouteng capsule	58 (0.12)	✓	✓
		Qinggan Jiangya capsule	5 (0.01)	✓	✓
	Soothe the liver and extinguish wind	Jiuqiang Naoliqing	8,689 (17.67)	✕	✕
		ShanJu Jiangya capsule	575 (1.17)	✕	✕
		Angong Jiangya pill	65 (0.13)	✕	✕
		Shanlvcha Jiangya tablet	6 (0.01)	✕	✕
Circulate blood and eliminate stasis formulas (162, 0.44)	Circulate blood and resolve stagnation	Xinkeshu capsule	104 (0.21)	✕	✕
	Transform stasis and soothe the chest	Qili Qiangxin capsule	45 (0.09)	✕	✕
		Xinmaitong tablet	7 (0.01)	✕	✕
		Naoxuekang capsule	6 (0.01)	✕	✕
Open the orifices formulas (317, 0.86)	Clear heat and open the orifices	Ershiwuwei Shanhu pill	313 (0.64)	✕	✕
		Annao pill	4 (0.01)	✕	✕
Calm the mind formulas (9, 0.02)	Tonify the spleen and kidney	Qishiwei Zhenzhu pill	9 (0.02)	✕	✕
Not clear (3736, 10.16)		Luobuma Jiangya tablet	1,823 (3.71)	✕	✕
		Duzhong Pinya tablet	949 (1.93)	✕	✕
		Duzhong Jiangya tablet	310 (0.63)	✕	✕
		Jiangya tablet	310 (0.63)	✕	✕
		Yangyin Jiangya capsule	96 (0.20)	✕	✕
		Changchunbao oral solution	78 (0.16)	✕	✕
		Tianma Shouwu capsule	47 (0.10)	✕	✕
		Duzhong granule	40 (0.08)	✕	✕
		Zhongjing Jiangya tablet	31 (0.06)	✕	✕
		Shanhu Qishiwei pill	19 (0.04)	✕	✕
		Gaoxueya Sujiang pill	9 (0.02)	✕	✕
		Luobu Maye tablet	7 (0.01)	✕	✕
		Shuxinning tablet	6 (0.01)	✕	✕
		Xinshubao capsule	4 (0.01)	✕	✕
		Juming Jiangya pill	2 (0.00)	✕	✕
		Xinnao Jiangya tablet	2 (0.00)	✕	✕
		Maijunan tablet	1 (0.00)	✕	✕
		Xingshen Jiangya tablet	1 (0.00)	✕	✕
		Yinaoning tablet	1 (0.00)	✕	✕

Note: ✓: Concordance with guideline recommendations; ✕: Discordant with guideline recommendations.

Only 1.86% (n = 14,642) of CPM prescriptions were concordant with guidelines. Among the 38 antihypertensive CPM, only 7 were recommended by guidelines, and these were primarily classified as treat wind formulas (Table 4). The most frequently prescribed antihypertensive CPM were Jiuqiang Naoliqing (n = 8,689, 17.67%) and Zhenju Jiangya tablet (n = 7,251, 14.74%), however, neither of them was recommended by the guidelines. Other commonly prescribed CPM were Qiangli Dingxuan tablet/capsule (n = 6,024, 12.25%), Qingnao Jiangya tablet (n = 5,348, 10.87%), and Niuhuang Jiangya tablet/pill (n = 2,996, 6.09%). These three CPM were recommended by the 2021 CPM guidelines for their TCM efficacy in soothing the liver and submerging yang. In contrast, several CPMs explicitly recommended by the 2021 CPM guidelines and the 2019 hypertension expert consensus were prescribed less frequently. These included Songling Xuemaikang capsule (n = 309, 0.63%), Tianma Gouteng capsule (n = 58, 0.12%), Qinggan Jiangya capsule (n = 5, 0.01%), and Xinmaitong tablet (n = 7, 0.01%).

**FIGURE 5:**
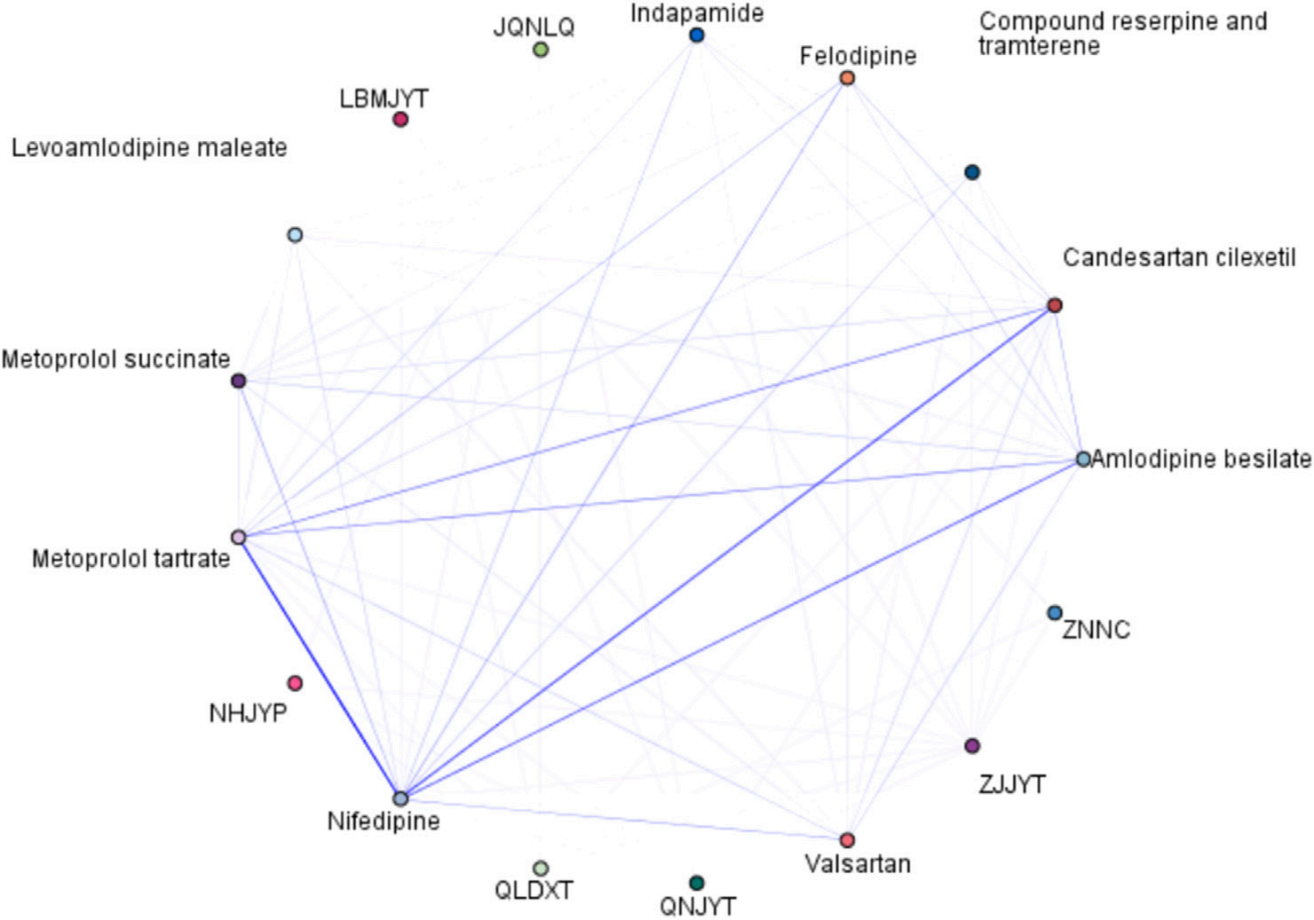
Association rule diagram of antihypertensive CPM and WM. Note: JQNLQ, Jiuqiang Naoliqing; ZJJYT, Zhenju Jiangya tablet; QLDXT, Qiangli Dingxuan tablet/capsule; QNJYT, Qingnao Jiangya tablet; NHJYP, Niuhuang Jiangya tablet/pill; LBMJYT, Luobuma Jiangya tablet; ZNNC, Zhennaoning capsule/granule; DZPYT, Duzhong Pinya tablet; SJJYC, ShanJu Jiangya capsule; ESWWSHP, Ershiwuwei Shanhu pill.

Among the antihypertensive CPM combinations, the combination of a single WM with a single CPM was the most common, while triple therapy of CPM was not observed (Figure 5; Table 5). The most frequently prescribed combination was Jiuqiang Naoliqing + nifedipine (*support* 17.15%, *confidence* 4.64%), followed by Zhenju Jiangya tablet + nifedipine (*support* 14.08%, *confidence* 5.07%), and Qiangli Dingxuan tablet/capsule + nifedipine (*support* 12.06%, *confidence* 3.64%). Among these combinations, only Qiangli Dingxuan tablet/capsule was recommended for use in combination with WM according to the guideline.

**TABLE 5:** Commonly prescribed antihypertensive CPM combination and guideline concordance.

Internet hospitals				Guidelines	
Prescribed CPM combination	Support (%)	Confidence (%)	Lift	2021 CPM guideline	2019 hypertension expert consensus
CPM combined with WM					
JQNLQ + A	17.15	4.64	0.81	✕	✕
JQNLQ + B	17.15	2.88	0.84	✕	✕
JQNLQ + C	17.15	2.6	0.88	✕	✕
ZJJYT+ A	14.08	5.07	0.89	✕	✕
ZJJYT + B	14.08	3.59	1.05	✕	✕
ZJJYT + D	14.08	2.51	1.26	✕	✕
ZJJYT + C	14.08	2.17	0.73	✕	✕
QLDXT + A	12.06	3.64	0.64	✓	✕
QLDXT+ C	12.06	2.77	0.94	✓	✕
QLDXT + B	12.06	2.48	0.73	✓	✕
CPM combined with CPM					
JQNLQ + QLDXT	8.65	0.44	0.06	✕	✕
JQNLQ + NHJYT	8.65	0.42	0.14	✕	✕
JQNLQ + QNJYT	8.65	0.32	0.06	✕	✕
QLDXT + QNJYT	7.23	0.29	0.05	✕	✕
QNJYT + NHJYT	5.33	0.34	0.11	✕	✕
QNJYT + DZPYT	5.33	0.24	0.26	✕	✕
NHJYT + QLDXT	2.99	0.4	0.06	✕	✕
NHJYT + LBMJYT	2.99	0.23	0.13	✕	✕
LBMJYT + QNJYT	1.8	0.5	0.09	✕	✕
LBMJYT + QLDXT	1.8	0.39	0.05	✕	✕

Note: JQNLQ, jiuqiang naoliqing; ZJJYT, zhenju jiangya tablet; QLDXT, Qiangli Dingxuan tablet/capsule; NHJYT, Niuhuang Jiangya tablet/pill; QNJYT, qingnao jiangya tablet; DZPYT, duzhong pinya tablet; LBMJYT, luobuma jiangya tablet; A, nifedipine; B, metoprolol tartrate; C, candesartan cilexetil; D, amlodipine besilate; ✓: Concordance with guideline recommendations; ✕: Discordant with guideline recommendations.

Two CPM combination prescriptions were not recommended in either of the guidelines (Table 5). The most frequently prescribed two-CPM combinations were Jiuqiang Naoliqing + Qiangli Dingxuan tablet/capsule (*support* 8.65%, *confidence* 0.44%)*, followed by* Jiuqiang Naoliqing + Niuhuang Jiangya tablet/pill (*support* 8.65%, *confidence* 0.42%),both of which are indicated fort hypertension with hyperactivity of liver yang pattern. Descriptions of the TCM syndromes are detailed in Supplementary Table S5. The third most common combination was Jiuqiang Naoliqing + Qingnao Jiangya tablet (*support* 8.65%, *confidence* 0.32%), which shared common components, including *Achyranthis bidentatae radix* and *Magentitum* (Supplementary Table S6).

## 4 Discussion

Hypertension is a leading health problem worldwide, and the prevention and treatment is very important (Yao et al., 2024). This study analyzed the prescribing characteristics and guideline concordance of antihypertensive WM and CPM across 787,209 hypertension visits in Internet hospitals in China. Our findings revealed that female patients were more likely to receive CPM alone compared to male patients, which is consistent with previous studies (Tsai et al., 2014; Xu et al., 2021a), reflecting women’s greater higher acceptance of TCM. Furthermore, hypertension patients aged 60 years or older were more frequently prescribed a combination of CPM and WM, primarily due to their higher prevalence of comorbidities and consequent need for comprehensive therapeutic strategies. Coronary heart disease was the most common comorbidity in our study, aligning with extensive evidence identifying hypertension as the primary contributor of CVD (Lu and Lan, 2022; Wang et al., 2022; GBD, 2019 Diseases and Injuries Collaborators, 2020). Patients with coronary heart disease were also more likely to prescribe combinations of CPM and WM, probably due to the complexity of their condition, which requires multi-targeted treatment. While antihypertensive WM target specific pathway (e.g., renin-angiotensin-aldosterone system blockade), CPM may provide complement effects through distinct mechanisms such as microcirculatory improvement or anti-inflammatory effects (Lauder et al., 2023; Jin et al., 2024). The combination may offer synergistic benefits, leading clinicians to prefer integrated treatment strategies.

### 4.1 Prescribing characteristics and guideline concordance of antihypertensive WM

Our findings on antihypertensive WM prescribing characteristics in Internet hospitals align with those from offline hospitals in China. A nationwide survey of primary healthcare sites reported nifedipine as the most prescribed individual medication, with CCBs, ARBs, and beta-blockers being the most frequently prescribed classes (Su et al., 2017). Similarly, an analysis of multiple offline hospitals indicated that CCBs and ARBs were the predominant classes, with diuretics + ARBs and diuretics + CCBs + ARBs being the most common two- and three-drug combinations, respectively (Yang et al., 2023). Consistent with these findings, our study identified CCBs, particularly nifedipine, as the most prescribed class in Internet hospitals, the most frequent dual therapy was nifedipine with metoprolol tartrate, while the predominant triple therapy combined candesartan cilexetil, nifedipine, and metoprolol tartrate. The consistent antihypertensive prescribing patterns in Internet hospitals, coupled with their enhanced accessibility and time- and cost-saving benefits, demonstrate their potential to support China’s hypertension management goals. This innovative healthcare delivery model effectively complements conventional care, particularly for long-term medication management in the growing hypertensive population in China.

The analysis revealed guideline concordance of antihypertensive WM in Internet hospitals is relatively satisfactory. All prescribed WM from the four first-line classes (ACEIs, ARBs, CCBs, and diuretics) were guideline-recommended. Furthermore, frequently prescribed classes such as SPCs, MRAs and beta-blockers are also recommended by the Chinese guideline. Although recommended as first-line options for hypertension management (McEvoy et al., 2024), ACEIs and diuretics showed lower prescription rates compared to SPCs in our study. This preference for SPCs may be attributed to their advantages in reducing pill burden and enhancing medication adherence and persistence. Consistent with our findings, previous research indicated that over 70% of clinicians preferentially prescribe SPCs as the first-line therapy for patients with stage 2 hypertension (Deng and Fan, 2024). Notably, our study identified frequent prescribing of dual CCBs therapy, with the nifedipine-amlodipine besylate combination representing the third most common dual therapy medications. However, dual CCBs therapy can cause ankle edema, headache, flushing and tachycardia (Jones et al., 2024). Current guidelines also recommend against combining medications with similar mechanisms of action due to insufficient high-quality evidence regarding the safety and efficacy of dual CCB therapy (Niu, 2018; Whelton et al., 2018). These findings underscore the necessity of enhanced physician education on appropriate antihypertensive combination therapies.

### 4.2 Prescribing characteristics and guideline concordance of antihypertensive CPM

The prescription of CPM should strictly adhere to the principle of “syndrome differentiation and treatment”, a cornerstone of TCM theory (Wang J. et al., 2014). Deviation from this principle may compromise the appropriateness of CPM prescriptions, potentially leading to suboptimal therapeutic outcomes and increased risks of adverse effects (Fan, 2017). However, in our study, very few prescriptions in Internet hospitals had diagnoses of TCM syndromes. This discrepancy may be attributed to several potential factors. First, syndrome differentiation requires a detailed and personalized assessment of the patient’s condition. However, online consultations limit clinicians' ability to fully utilize the four diagnostic TCM methods (observation, auscultation, inquiry and palpation), which are essential for accurate syndrome identification. Second, most healthcare providers in Internet hospitals are trained in WM and often lack sufficient knowledge or proficiency in TCM syndrome differentiation (Luo et al., 2017; Liu et al., 2017). This gap in expertise may result in prescriptions that do not align with TCM principles. To address these challenges, it is essential to enhance the education of WM practitioners in TCM theory and encourage them to obtain TCM-related qualifications. Moreover, to overcome the limitations of online consultations, Internet hospitals should introduce Artificial Intelligence (AI)-enabled TCM diagnostic systems, such as tongue and pulse diagnostic devices. These technologies can replicate the traditional TCM diagnostic methods of observation and palpation, enabling more accurate identification of TCM syndromes. Integrating these systems into online platforms would significantly enhance the ability to perform TCM syndrome differentiation remotely.

A significant finding of this study is the suboptimal utilization of guideline-recommended antihypertensive CPMs in Internet hospitals. Among the 38 prescribed CPM, only 7 were recommended by guidelines. This discrepancy highlights a substantial gap between clinical practice in Internet hospitals and evidence-based recommendations in guidelines. A notable example is Jiuqiang Naoliqing, the most frequently prescribed CPM in our study, which is not recommended by hypertension guidelines (Xu F. Q., 2022; Wang J. G., 2024). This CPM exerts therapeutic effects by soothing the liver, submerging yang hyperactivity, and clearing liver fire. Its primary component, achyranthis bidentatae radix, has demonstrated significant reductions in liver yang hyperactivity symptoms, blood pressure, and cardiac remodeling in preclinical studies using hypertensive rat models (Wu et al., 2024; Zhang et al., 2024b). However, the absence of robust randomized controlled trials (RCTs) evaluating clinical endpoints, such as blood pressure control rates, cardiovascular event reduction, or long-term safety profiles, precludes its inclusion in evidence-based guidelines. This evidence gap underscores the critical need for rigorous RCTs with standardized TCM syndrome differentiation criteria to validate the therapeutic efficacy and safety of widely utilized CPM like Jiuqiang Naoliqing. Furthermore, clinical guidelines should be regularly updated to systematically incorporate emerging clinical efficacy evidence, ensuring alignment between guideline-recommended CPM and advancing evidence.

Furthermore, duplicate prescriptions of CPM for the same TCM syndrome in Internet hospitals often deviate from guidelines recommendations (State Administration of Traditional Chinese Medicine, 2010; Society of Cardiovascular Diseases, 2019). For instance, Jiuqiang Naoliqing was frequently combined with Qiangli Dingxuan tablet/capsule, both of which are indicated for hypertension with a pattern of hyperactivity of liver yang. However, there is no evidence to suggest that combining these two CPM enhances their clinical effectiveness in treating hypertension. On the contrary, such duplicate prescriptions may lead to overdosing and reduced therapeutic effectiveness (Xiong et al., 2013). A common example in our study was the combination of Jiuqiang Naoliqing and Qingnao Jiangya tablet, both of which contain *Achyranthis bidentatae radix* and *Magentitum*. This combination may increase the risk of adverse effects, including transient hypotension, hyperuricaemia, and elevated blood glucose levels (Xiong et al., 2013; Yu H. et al., 2019; Wang and Liu, 2025). To address these issues, implementing a Clinical Decision Support System (CDSS) in Internet hospitals is recommended. A CDSS integrates patient data with guideline recommendations, ensuring evidence-based prescribing. Previous studies in China have demonstrated its effectiveness in promoting guideline-adherent antihypertensive treatment (Lu et al., 2022; Yu et al., 2023). The CDSS should be regularly updated to include CPM-specific features, such as active components, TCM indications, and alerts for risks like overdosing, drug-drug interactions, or dual therapy. By leveraging such a system, Internet hospitals can improve the appropriateness and safety of CPM prescriptions, ultimately improving outcomes for hypertension patients.

### 4.3 Recommendations for antihypertensive medication management in Internet hospitals

To address the identified discrepancies and strengthen regulation of antihypertensive CPM management in the treatment of hypertension in Internet hospitals, the following recommendations are proposed:

(1) Integrate AI and CDSS for TCM precision: Implement AI-enabled TCM diagnostic tools (e.g., tongue and pulse analyzers) to improve TCM syndrome identification online and provide personalized therapy for hypertension patients, and integrate a CDSS to prevent risks such as duplicate therapies or drug interactions, thereby enhancing real-time regulatory oversight of Internet hospitals.
(2) Enhance TCM education and guideline concordance: Provide training for WM clinicians on TCM theory and syndrome differentiation to bridge the gap between WM and TCM practices, and strengthen their adherence to clinical guidelines to ensure patients receive the most effective and evidence-based TCM treatments, thereby improving the quality of healthcare services in Internet hospitals.
(3) Conduct high-quality research and update guidelines: Perform RCT to evaluate the efficacy and safety of commonly prescribed antihypertensive CPM, and guidelines should be regularly updated to incorporate new evidence, including TCM syndrome differentiation principles, ensuring alignment between guideline-recommended CPM and advancing evidence.

### 4.4 Limitations

This study has several limitations. First, the scarcity of TCM syndrome diagnoses in the prescriptions precluded a comprehensive assessment of the underlying rationales for clinicians' selection of specific CPM, making it impossible to determine whether the choices were based on TCM syndrome differentiation. Second, the data for this study were obtained from the YIMRP. Although this platform encompasses around 80% of enterprise-led Internet hospitals in China, it may not comprehensively represent the prescribing characteristics across all regions and Internet hospitals nationwide. Third, although this study provides comprehensive analysis of prescribing characteristics and guideline concordance, the lack of clinical outcome data (e.g., blood pressure control rates, cardiovascular events, or adverse effects) in our database precluded assessment of treatment effectiveness and the impact of guideline concordance on patient outcomes. Future research should incorporate outcome data to provide more actionable insights into antihypertensive medication management in this emerging healthcare model.

## 5 Conclusion

The prescribing characteristics of antihypertensive WM in Internet hospitals closely align with those in offline hospitals in China, demonstrating relatively satisfactory guideline concordance and highlighting their potential to complement conventional care for hypertension management. However, some issues persist in the prescription of antihypertensive CPM, including the lack of TCM syndrome differentiation, frequent prescription of non-guideline-recommended CPM, and duplicate therapies. It is crucial to strengthen the management of CPM in Internet hospitals to further enhance their role in optimizing hypertension care.

## Data Availability

The raw data supporting the conclusions of this article will be made available by the authors, without undue reservation.

## Glossary

ACC/AHA: American College of Cardiology and the American Heart Association
ACEIs: Angiotensin-converting enzyme inhibitors
AI: Artificial intelligence
ANOVA: Analysis of variance
ARBs: Angiotensin receptor blockers
ARNI: Angiotensin receptor-neprilysin inhibitor
ATC: Anatomical therapeutic chemical
CCBs: Calcium channel blockers
CDSS: Clinical decision support system
CPM: Chinese patent medicine
CVD: Cardiovascular disease
DALYs: Disability-adjusted life years
DZPYT: Duzhong Pingya tablet
ESC/ESH: European Society of Cardiology and European Society of hypertension
ESWWSHP: Ershiwuwei Shanhu pill
ICD-10: International classification of diseases, version 10
JQNLQ: Jiuqiang Naoliqing
LBMJYT: Luobuma Jiangya tablet
MRAs: Mineralocorticoid receptor antagonists
NHJYP: Niuhuang Jiangya tablet/pill
QLDXT: Qiangli Dingxuan tablet/capsule
QNJYT: Qingnao Jiangya tablet
RCTs: Randomized controlled trials
SD: Standard deviation
SJJYC: Shanju Jiangya capsule
SPCs: Single-pill combinations
STROBE: Strengthening the reporting of observational studies in epidemiology
TCM: Traditional Chinese medicine
WM: Western medicine
YIMRP: Yinchuan internet medical regulatory platform
ZJJYT: Zhenju Jiangya tablet
ZNNC: Zhennaoning capsule/granule
