# Supplementary material for: Prescribing characteristics and guideline concordance of antihypertensive western and Chinese patent medicine in Internet hospitals in China: a cross-sectional study
Source: Front Pharmacol. 2025 Apr 28;16:1580787. doi: 10.3389/fphar.2025.1580787 (PMC12076013; doi:10.3389/fphar.2025.1580787)
Supplement: Supplementary file 1 [file DataSheet1.docx]

Supplementary Table S1. Guideline-recommended antihypertensive WM

| Guidelines | Class | Recommended individual WM |
| --- | --- | --- |
| 2017 ACC/AHA guideline | First-line antihypertensive WM | |
|  | ACEIs | Benazepril, Captopril, Enalapril, Fosinopril, Lisinopril, Moexipril, Perindopril, Quinapril, Ramipril, Trandolapril |
|  | ARBs | Azilsartan, Candesartan, Eprosartan, Irbesartan, Losartan, Olmesartan, Telmisartan, Valsartan |
|  | CCBs—dihydropyridine | Amlodipine, Felodipine, Isradipine, Nicardipine SR, Nifedipine LA, Nisoldipine |
|  | CCBs—nondihydropyridine | Diltiazem ER, Verapamil IR, Verapamil SR, Verapamil delayed-onset ER |
|  | Thiazide or thiazide-type diuretics | Chlorthalidone, Hydrochlorothiazide, Indapamide, Metolazone |
|  | Second-line antihypertensive WM | |
|  | Diuretics—loop | Bumetanide, Furosemide, Torsemide |
|  | Diuretics—potassium sparing | Amiloride, Triamterene |
|  | Diuretics—aldosterone antagonists | Eplerenone, Spironolactone |
|  | Beta blockers—cardioselective | Atenolol, Betaxolol, Bisoprolol, Metoprolol tartrate, Metoprolol succinate |
|  | Beta blockers—cardioselective and vasodilatory | Nebivolol |
|  | Beta blockers—noncardioselective | Nadolol, Propranolol IR, Propranolol LA |
|  | Beta blockers—intrinsic sympathomimetic activity | Acebutolol, Penbutolol, Pindolol |
|  | Beta blockers—combined alpha and beta receptor | Carvedilol, Carvedilol phosphate, Labetalol |
|  | Renin inhibitor | Aliskiren |
|  | Alpha-1 blockers | Doxazosin, Prazosin, Terazosin |
|  | Central alpha2-agonist and other centrally acting drugs | Clonidine oral, Clonidine patch, Methyldopa, Guanfacine |
|  | Direct vasodilators | Hydralazine, Minoxidil |
| 2018 ESC/ESH guideline | First-line antihypertensive WM | |
|  | ACEIs |  |
|  | ARBs |  |
|  | Beta blockers |  |
|  | CBBs |  |
|  | Thiazides and thiazide-type diuretics | chlorthalidone, Indapamide |
|  | Second-line antihypertensive WM | |
|  | Alpha-blockers |  |
|  | Centrally acting drugs |  |
| 2018 Chinese guideline | First-line antihypertensive WM | |
|  | ACEIs | Captopril, Enalapril, Benazepril, Lisinopril, Ramipril, Fosinopril, Cilazapril, Perindopril, Imidapril |
|  | ARBs | Losartan, Valsartan, Irbesartan, Telmisartan, Candesartan, Olmesartan, Allisartan isoproxil |
|  | Beta blockers | Bisoprolol, Metoprolol, Metoprolol succinate, Atenolol, Propranolol, Betaxolol |
|  | CCBs—dihydropyridine | Nifedipine, Amlodipine, Levamlodipine, Felodipine, Lacidipine, Nicardipine, Nitrendipine, Benidipine, Lercanidipine, Manidipine, Cilnidipine, Barnidipine |
|  | CCBs—nondihydropyridine | Verapami, Diltiazem |
|  | Thiazides and thiazide-type diuretics | Hydrochlorothiazide, Chlorthalidone, Indapamide |
|  | Second-line antihypertensive WM | |
|  | Alpha-blockers | Doxazosin, Prazosin, Terazosin |
|  | Alpha and beta-blockers | Labetalol, Carvedilol, Arotinolol |
|  | Centrally acting agents | Reserpine, Clonidine, Clonidine patch, Methyldopa |
|  | Direct vasodilators | Minoxidil, Hydralazine |
|  | MRAs | Spironolactone, Eplerenone |
|  | Renin inhibitors | Aliskiren |
|  | SPCs | Losartan Potassium/Hydrochlorothiazide, Valsartan/Hydrochlorothiazide, Irbesartan/Hydrochlorothiazide, Telmisartan/Hydrochlorothiazide, Olmesartan/Hydrochlorothiazide, Captopril/Hydrochlorothiazide, Lisinopril/Hydrochlorothiazide, Enalapril/Hydrochlorothiazide, Benazepril/Hydrochlorothiazide, Perindopril/Indapamide, Perindopril/Amlodipine, Perindopril Arginine/Amlodipine Besylate, Amlodipine/Valsartan, Amlodipine/Telmisartan, Amlodipine/Benazepril, Amiloride/Hydrochlorothiazide, Nitrendipine/Atenolol, Compound Reserpine Tablets, Compound Reserpine-Triamterene Tablets, Enalapril/Folic Acid, Amlodipine/Atorvastatin, Candesartan Cilexetil/Hydrochlorothiazide |
|  | Diuretics—loop | Furosemide, Torasemide |
|  | Diuretics—potassium sparing | Amiloride, Triamterene |

Supplementary Table S2. Guideline-recommended antihypertensive CPM and their recommended usage

| Guidelines | TCM categories | Recommended individual CPM | Recommended usage |
| --- | --- | --- | --- |
| 2021 CPM guideline | Hyperactivity of liver yang pattern | Songling Xuemaikang capsule | Combined with WM |
|  |  | Tianma Gouteng granule | Combined with WM |
|  |  | Qiangli Dingxuan tablet | Combined with WM |
|  |  | Qingnao Jiangya tablet | Monotherapy or combined with WM |
|  | Heart and liver pattern, Phlegm fire disturbing the heart pattern | Niuhuang Jiangya pill | Monotherapy or combined with WM |
|  | Upward flaming of liver  fire pattern, Yin deficiency of the  liver and kidney pattern | Qinggan Jiangya capsule | Combined with WM |
|  | Upward flaming of liver  fire pattern | Fufang Luobuma granule | Combined with WM |
| 2019 hypertension expert consensus | Hyperactivity of liver yang pattern | Tianma Gouteng granule |  |
|  |  | Qinggan Jiangya capsule |  |
|  |  | Songling Xuemaikang |  |
|  | Phlegm dampness accumulating in the  spleen pattern | Banxia Tianma pill |  |
|  | Deficiency of kidney yin and yang pattern | Liuwei Dihuang pill |  |
|  |  | Jiju Dihuang pill |  |
|  |  | Jinkui Shenqi pill |  |
|  | Other | Yangxue Qingnao granule |  |

Supplementary Table S3. Individual and class of non-first-line antihypertensive WM in Internet hospitals and guideline concordance

| Internet hospitals | | |  | Guidelines | | |
| --- | --- | --- | --- | --- | --- | --- |
| Class | Individual WM | Prescription frequency (n, %) |  | 2017 ACC/AHA guideline | 2018 ESC/ESH guideline | 2018 Chinese guideline |
| ARNI | Sacubitril valsartan sodium | 344 (0.04) |  | × | × | × |
| Direct Vasodilators | Dibazole | 333 (0.04) |  | × | × | × |
| MRAs | Spirolactone | 8,437 (1.07) |  | × | × | √ |
| SPCs | Compound reserpine and tramterene | 29,787 (3.78) |  | × | × | × |
|  | Irbesartan and hydrochlorothiazide | 19,824 (2.52) |  | × | × | √ |
|  | Compound reserpine | 11,616 (1.48) |  | × | × | √ |
|  | Aspartic acid amlodipine | 7,723 (0.98) |  | × | × | × |
|  | Valsartan and hydrochlorothiazide | 5,589 (0.71) |  | × | × | √ |
|  | Valsartan and amlodipine | 4,405 (0.56) |  | × | × | √ |
|  | Compound bendazol and hydrochlorothiazide | 4,378 (0.56) |  | × | × | × |
|  | Losartan potassium and hydrochlorothiazide | 2,806 (0.36) |  | × | × | √ |
|  | Compound captopril | 691 (0.09) |  | × | × | × |
|  | Enalapril maleate and folic acid | 629 (0.08) |  | × | × | √ |
|  | Perindopril and indapamide | 577 (0.07) |  | × | × | √ |
|  | Amlodipine and benazepril | 355 (0.05) |  | × | × | √ |
|  | Compound amiloride hydrochloride | 295 (0.04) |  | × | × | √ |
|  | Dual-triazine compound reserpine | 184 (0.02) |  | × | × | √ |
|  | Amlodipine and atorvastatin calcium | 156 (0.02) |  | × | × | √ |
|  | Olmesartan medoxomil and Hydrochlorothiazide | 136 (0.02) |  | × | × | × |
|  | Telmisartan and hydrochlorothiazide | 55 (0.01) |  | × | × | √ |
|  | Perindopril and amlodipine | 53 (0.01) |  | × | × | √ |
|  | Benazepril and hydrochlorothiazide | 16 (0.00) |  | × | × | √ |
|  | Compound reserpine and hydrochlorthiazide | 4 (0.00) |  | × | × | √ |
|  | Enalapril and hydrochlorothiazide | 3 (0.00) |  | × | × | √ |
| Alpha and beta-blockers | Labetalol | 1,365 (0.17) |  | √ | × | √ |
|  | Carvedilol | 1,298 (0.16) |  | √ | × | √ |
|  | Arotinolol hydrochloride | 789 (0.10) |  | × | × | √ |
|  | Bevantolol hydrochloride | 1 (0.00) |  | × | × | × |
| Alpha-blockers | Terazosin hydrochloride | 3,773 (0.48) |  | √ | √ | √ |
|  | Doxazosin mesylate | 353 (0.04) |  | √ | √ | √ |
|  | Urapidil | 1 (0.00) |  | × | × | × |
| Beta-blockers | Metoprolol tartrate | 101,689 (12.92) |  | √ | √ | √ |
|  | Metoprolol succinate | 38,494 (4.89) |  | √ | √ | √ |
|  | Bisoprolol fumarate | 22,000 (2.79) |  | √ | √ | √ |
|  | Propranolol hydrochloride | 7,739 (0.98) |  | √ | √ | √ |
|  | Atenolol | 1,252 (0.16) |  | √ | √ | √ |

Note: √: Concordance with guideline recommendations; ×: Discordant with guideline recommendations; ARNI, angiotensin receptor-neprilysin inhibitor; MRAs, mineralocorticoid receptor antagonists; SPCs, single-pill combinations.

Supplementary Table S4. Pinyin and Chinese term of 38 antihypertensive CPM

| Pinyin term | Chinese term | Pinyin term | Chinese term |
| --- | --- | --- | --- |
| Angong Jiangya pill | 安宫降压丸 | Qingnao Jiangya tablet | 清脑降压片 |
| Annao pill | 安脑丸 | ShanJu Jiangya capsule | 山菊降压胶囊 |
| Duzhong Jiangya tablet | 杜仲降压片 | Shanlvcha Jiangya tablet | 山绿茶降压胶囊 |
| Duzhong granule | 杜仲颗粒 | Shanhu Qishiwei pill | 珊瑚七十味丸 |
| Duzhong Pinya tablet | 杜仲平压片 | Shuxinning tablet | 舒心宁片 |
| Ershiwuwei Shanhu pill | 二十五味珊瑚丸 | Songling Xuemaikang capsule | 松龄血脉康胶囊 |
| Gaoxueya Sujiang pill | 高血压速降丸 | Tianma Gouteng capsule | 天麻钩藤胶囊 |
| Jiangya tablet | 降压片 | Tianma Shouwu capsule | 天麻首乌胶囊 |
| Jiuqiang Naoliqing | 久强脑立清 | Xinkeshu capsule | 心可舒胶囊 |
| Juming Jiangya pill | 菊明降压丸 | Xinmaitong tablet | 心脉通片 |
| Luobuma Jiangya tablet | 罗布麻降压片 | Xinnao Jiangya tablet | 心脑降压片 |
| Luobu Maye tablet | 罗布麻叶片 | Xinshubao capsule | 心舒宝胶囊 |
| Maijunan tablet | 脉君安片 | Xingshen Jiangya tablet | 醒神降压片 |
| Naoxuekang capsule | 脑血康胶囊 | Yangyin Jiangya capsule | 养阴降压胶囊 |
| Niuhuang Jiangya tablet/pill | 牛黄降压片/丸 | Yinaoning tablet | 益脑宁片 |
| Qishiwei Zhenzhu pill | 七十味珍珠丸 | Changchunbao oral solution | 长春宝口服液 |
| Qili Qiangxin capsule | 芪苈强心胶囊 | Zhenju Jiangya tablet | 珍菊降压片 |
| Qiangli Dingxuan tablet/capsule | 强力定眩片/胶囊 | Zhennaoning capsule/granule | 镇脑宁胶囊/颗粒 |
| Qinggan Jiangya capsule | 清肝降压胶囊 | Zhongjing Jiangya tablet | 仲景降压片 |

Supplementary Table S5. Descriptions of TCM categories and syndromes

| English term | Chinese term | English definition/description |
| --- | --- | --- |
| TCM categories |  |  |
| Calm the mind formulas | 安神剂 | Formulas mainly consisting of medicines to calm the mind. Indicated for mental restlessness. These formulas are categorized into two types: formulas to calm the mind with heavy medicines; and formulas to tranquilize the mind by nourishing yin and blood |
| Circulate blood and eliminate stasis formulas | 祛瘀剂 | Formulas mainly consisting of blood-circulating medicines. They are indicated for blood stasis pattern |
| Open the orifices formulas | 开窍剂 | Formulas containing aromatic medicines. They are indicated for loss of consciousness and blockage of orifices. These formulas are categorized into two types: formulas to clear heat; and formulas to warm the interior and open the orifices |
| Treat wind formulas | 治风剂 | Formulas mainly consisting of wind-removing or wind-extinguishing medicines. They are indicated for external or internal wind |
| TCM syndromes |  |  |
| Deficiency of the liver and kidney pattern | 肝肾两虚证 | Characterized by pale, lustreless complexion, pale lips and fingernails, dizziness, tinnitus, dry eyes, palpitations, insomnia, dream-disturbed sleep, jumpiness or easily startled, irregular menstruation, scanty menstrual volume, amenorrhoea and low back soreness. The tongue is red. The pulse is thready. This pattern often results from chronic diseases or age-related liver blood/kidney essence deficiency |
| Fire hyperactivety of the heart and liver pattern | 心肝火旺证 | Characterized by fever, thirst, restlessness, irritability, red face and eyes, pain in the subcostal region, a bitter mouth, insomnia and dreamdisturbed sleep. The tongue is red with a yellow coating. The pulse is rapid and forceful. This pattern often occurs when exuberant fire/heat affects the heart and liver |
| Hyperactivity of liver yang pattern | 肝阳上亢证 | Characterized by dizziness, blurred vision, tinnitus, lower back pain, limb numbness, feverish sensations in the palms, soles and chest, flushed cheeks, restlessness, irritability, and a dry and bitter mouth. The tongue is red with a scanty coating. The pulse is thready and rapid. This pattern often occurs when liver yin fails to control liver yang |
| Internal stirring of liver wind pattern | 肝风内动证 | Characterized by convulsions of the four limbs, vertigo and tremor. This pattern often results from wind yang, fire heat and yin blood deficiency |
| Kidney yin deficiency pattern | 肾阴亏虚证 | Characterized by low back/knee soreness and weakness, lassitude, dizziness, blurred vision, deafness, tinnitus, nocturnal emissions, a dry mouth, sore throat, flushed cheeks, feverish sensations in the palms, soles and chest, and tidal fever in the afternoon. The tongue is red with no or scanty coating. The pulse is thready and rapid. Often results from kidney yin dificiency |
| Phlegm dampness accumulating in the  spleen pattern | 痰湿中阻证 | Characterized by a greasy taste, a poor appetite, nausea, vomiting, abdominal masses/distension, splashing sounds in the stomach and loose stools. The tongue is pale and enlarged with a white, greasy coating. The pulse is soft and slack. This pattern often occurs when phlegm dampness obstructs the stomach and intestines |
| Phlegm fire disturbing the heart pattern | 痰火扰心证 | Characterized by hyperactivity, talkativeness, restlessness, impulsiveness, uncontrollable behaviour, inattention, feverish sensation in the chest, vexation, a poor appetite, a bitter mouth, constipation, and dark-yellow urine. The tongue is red with a yellow, greasy coating. The pulse is slippery and rapid. This pattern often occurs as a result of an internal build-up of phlegm fire |
| Stagnant blood obstructing the meridians pattern | 瘀血阻络证 | Characterized by a stabbing pain with a fixed location. Alternatively, purple macules, lumps or bleeding of dark blood may be present. The tongue is purple or with petechiae. The pulse is hesitant. This pattern often occurs when stagnant blood obstructs the meridians |
| Upward flaming of liver fire pattern | 肝火上炎证 | Characterized by fever, thirst, restlessness, insomnia, and headache. Alternatively, eye  redness, swelling and pain, sudden tinnitus/deafness, haematemesis, nosebleed and a red  face may be present. The tongue is red with a yellow coating. The pulse is wiry and rapid. This pattern often occurs when exuberance liver fire flows upward |
| Yin deficiency of the liver and kidney pattern | 肝肾阴虚证 | Characterized by dizziness, tinnitus, feverish sensations in the palms, soles and chest, a lowgrade fever, flushed cheeks, pain in the subcostal region, and low back/knee soreness and weakness. The tongue is red with a scanty coating. The pulse is thready and rapid. This pattern often occurs when yin deficiecy of the liver and kidney causes deficiency heat |
| Yin deficiency-induced yang hyperactivity pattern | 阴虚阳亢证 | Yin deficiency-induced yang hyperactivity pattern Characterized by tidal fever, night sweats, flushed cheeks, dizziness, blurred vision, restlessness, and insomnia. The tongue is dry and red. The pulse is thready and rapid. Often occurs when yin fluids fails to restrain yang |

Supplementary Table S6. The components, TCM syndrome and efficacy of the top 10 antihypertensive CPM

| CPM | Components (Latin names^f^) | TCM syndrome | TCM efficacy | Prescription frequency (n, %) |
| --- | --- | --- | --- | --- |
| Jiuqiang Naoliqing^a^ | *Magnetitum, Haematitum, Achyranthis bidentatae radix, Pinelliae Rhizoma praeparatum cum alumine, Chang rtzi, I-menthol, Borneolum syntheticum, Suis fellis pulvis, Cinnabaris* | Hyperactivity of liver yang pattern | Soothe the liver and submerge yang, clear and reduce liver fire | 8,689 (23.63) |
| Zhenju Jiangya tablet^b^ | *Chryanthemi indici flos, Margarita, Clonidine hydrochloride, Hydrochlorothiazide, Sophora japonica L* | Hyperactivity of liver yang pattern | Soothe the liver and submerge yang | 7,251 (19.72) |
| Qiangli Dingxuan tablet/capsule | *Gastrodiae rhizoma, Eucommiae cortex, Chryanthemi indici flos, Eucommiae folium, Chuanxiong Rhizoma* | Hyperactivity of liver yang pattern | Soothe the liver and submerge yang | 6,024 (16.38) |
| Qingnao Jiangya tablet^b^ | *Scutellariae radix, Prunellae spica, Flos sophorae immaturus, Magnetitum, Achyranthis bidentatae radix, Angelicae sinensis radix, Rehmanniae radix, Salviae miltiorrhizae radix et rhizoma, Hirudo, Uncariae ramulus cum uncis, Cassiae semen, Pheretima, Margaritifera concha* | Hyperactivity of liver yang pattern | Soothe the liver and submerge yang | 5,348 (14.55) |
| Niuhuang Jiangya tablet/pill^b^ | *Saigae tataricae cornu, Powerdered buffalo horn extract extract, Borneolum syntheticum, Scutellaria extract, Menthae haplocalycis herba, Margarita, Cassiae semen, Bovis calculus artifactus, Paeoniae radix alba, Scutellariae radix, Chuanxiong rhizoma, Nardostachyos radix et rhizoma, Curcumae radix.* | Fire hyperactivity of the heart and liver pattern | Soothe the liver and submerge yang, clear heat and transform phlegm, nourish the heart and calm the mind | 2,996 (8.15) |
| Luobuma Jiangya tablet^c^ | *Apocynum venetum, Prunellae spica, Uncariae ramulus cum uncis, Alismatis rhizoma, Margaritifera concha, Achyranthis bidentatae radix, Crataegi fructus, Chrysanthemi flos* | Hyperactivity of liver yang pattern, stagnant blood obstructing the meridians pattern | Soothe the liver and submerge yang, stop wind and relieve convulsions, circulate blood and alleviate pain | 1,823 (3.71) |
| Zhennaoning capsule^b^ | *Medulla sus domestica, Asari radix et rhizoma, Salviae miltiorrhizae radix et rhizoma, Powerdered buffalo horn extract extract, Chuanxiong Rhizoma, Gastrodiae rhizoma, Puerariae lobatae radix, Ligustici rhizoma et radix, Angelicae dahuricae radix* | Internal stirring of liver wind pattern | Soothe the liver and extinguish wind | 1,218 (4.96) |
| Duzhong Pinya tablet^c^ | *Eucommiae folium* | Deficiency of the liver and kidney pattern | Soothe the liver and submerge yang, strengthen the sinews and bones | 949 (2.58) |
| Shanju Jiangya capsule^d^ | *Crataegi fructus, Prunellae spica, Chrysanthemi flos, Cirsii herba, Aliamatis rhizoma, Cassiae semen* | Yin deficiency-induced yang hyperactivity pattern | Soothe the liver and submerge yang | 575 (1.56) |
| Ershiwuwei Shanhu pill^e^ | *Corrallium, Margarita, Lapis lazuli, Margaritifera concha, Chebulae fructus, Aucklandiae radix, Carthami flos, Caryophylli flos, Corallium japonicum kishinouye, Margarita, Lapis lazuli, Margaritifera concha, Chebulae frructus, Aucklandiae radix, Carthami flos, Caryophylli flos, Aquilariae lignum resinatum, Cinnabaris, Os Draconis, Calamina, Magnetitum, Sesamum indicum, Cucurbita, Aster tataricus, Swertia bimaculata, Acori calami rhizoma, Aconitum pendulum busch, Pyrethrum tatsienense, Glycyrrhizae radix et rhizoma, Croci stigma, Moschus Gypsum fibrosum, Limonitum* | Stagnant blood obstructing the meridians pattern | Harmonize meridians | 313 (0.85) |

Note: ^a^ Refer to Drug Standard of the Ministry of Health of the People's Republic of China, Chinese medicine prescription preparation, Volume 20; ^b^Refer to Chinese Pharmacopoeia, 2020 Edition Part 1; ^c^Refer to Drug Standard of the Ministry of Health of the People's Republic of China, Chinese medicine prescription preparation,Volume 17; ^d^Refer to State Drug Administration Standards (2001); ^e^Refer to Chinese Pharmacopoeia, 2015 Edition Part 1; ^f^The Latin names standardized by Chinese Pharmacopoeia (2020 edition).
